# Positive interactions and active mentalizing facilitate inter-brain synchronization in couples

**DOI:** 10.1093/scan/nsag083

**Published:** 2026-10-03

**Authors:** Isabella Schneider, Quentin Moreau, Daniel Neudecker, Sabine C Herpertz, Guillaume Dumas

**Affiliations:** Department of General Psychiatry, Center for Psychosocial Medicine, Heidelberg University, Heidelberg 69115, Germany; Federal Institute for Drugs and Medical Devices, Bonn 53175, Germany; Precision Psychiatry and Social Physiology Laboratory (PPSP), CHU Sainte-Justine Azrieli Research Center, Montréal, QC H3T 1C5, Canada; Department of Psychiatry, University of Montréal, Montréal, QC H2S 3H1, Canada; Department of General Psychiatry, Center for Psychosocial Medicine, Heidelberg University, Heidelberg 69115, Germany; Department of General Psychiatry, Center for Psychosocial Medicine, Heidelberg University, Heidelberg 69115, Germany; Precision Psychiatry and Social Physiology Laboratory (PPSP), CHU Sainte-Justine Azrieli Research Center, Montréal, QC H3T 1C5, Canada; Department of Psychiatry, University of Montréal, Montréal, QC H2S 3H1, Canada; Mila—Quebec AI Institute, Montréal, QC H2S 3H1, Canada

**Keywords:** hyperscanning, inter-brain synchronization, alignment, theory of mind, social interaction

## Abstract

High-quality interactions rely on socio-cognitive abilities, which help partners understand and respond to one another. Hyperscanning, which measures brain activity in multiple individuals simultaneously, can examine inter-brain synchronization as a possible signature of social coordination. In this study, we explore this synchronization during natural, everyday interactions in romantic couples. Forty-two dyads participated in a naturalistic behavioral paradigm while electroencephalography was recorded. We measured behavioral dyadic reciprocity and negative states, and computed inter-brain synchronization using the circular correlation coefficient (CCorr) across multiple frequency bands (theta [4-8 Hz], alpha [8-13 Hz], and beta [14-31 Hz]). Positive interactions elicited greater dyadic reciprocity and fewer negative states compared to negative interactions. At the neural level, inter-brain synchronization in the beta band was significantly higher during positive compared to negative interactions. Mentalizing compared to counting elicited increased inter-brain synchronization in both alpha and beta bands, indicating that active sociocognitive processing enhances neural alignment between partners. These findings demonstrate that inter-brain dynamics provide a neural signature of both interaction valence and mentalizing engagement during real-world social interactions, putting forward inter-brain synchronization as a sensitive marker of sociocognitive coordination.

## Introduction

Successful interpersonal interactions depend on complex sociocognitive abilities, including the capacity to attribute mental and affective states to others, a process known as mentalizing (Dunbar and Shultz 2007, Cacioppo *et al*. 2014, Ayache *et al*. 2024). This capacity is especially critical in romantic relationships, where individual differences in mentalizing predict relationship satisfaction and stability (Hazan and Shaver 1987). Interaction quality determines relationship outcomes: happy couples exhibit a higher ratio of positive to negative interactions (Williams 1979), while negative behaviors such as withdrawal and defensiveness predict relationship deterioration (Gottman and Krokoff 1989).

To better understand the neural underpinnings of such complex and dynamic social interactions, especially in close relationships, neuroscience offers valuable tools. Yet, until recently, studies in social neuroscience have mainly focused on studying single individuals passively observing social stimuli (Schilbach *et al*. 2013). Over the past decade, new approaches have emerged, with an increased focus on studying interactional processes at the dyadic and dynamic levels [see Redcay and Schilbach (2019) for a review]. These more naturalistic methods have proposed bio-behavioral synchronization as an emergent phenomenon arising from dyadic coordination (Feldman 2012, Dumas and Fairhurst 2021). Synchronization is the rhythmic adaptation of behavior, physiology, and brain activity over the time of interaction. It can be based on the unconscious, individual perception of multiple stimuli (e.g. facial expressions, gestures, intonation), their regularity and familiarity (Hoehl *et al*. 2021), as well as internal synchronizers like personality traits or motivational states (Dikker *et al*. 2021). The result is a mutual adaptation to stimuli and an alignment of mental states between members of the interaction (Hoehl *et al*. 2021). Studies indicate that people are selective in their synchrony depending on, among other things, sympathy, familiarity of the interaction partners, and valence of the interaction (Rennung and Göritz 2016, Djalovski *et al*. 2021, Hoehl *et al*. 2021). The potential benefits of synchronization may lie in increased efficiency and performance (e.g. through increased predictability of the other person’s behavior), as well as the strengthening of social bonding and prosocial behavior (Rennung and Göritz 2016, Djalovski *et al*. 2021, Hoehl *et al*. 2021).

At the neural level, inter-brain synchronization (IBS) can be investigated through hyperscanning, i.e. the simultaneous recording of neuronal activity in two or more individuals (Montague *et al*. 2002, Dumas *et al*. 2010). Functional near-infrared spectroscopy (Nozawa *et al*. 2016) or functional magnetic resonance imaging (Montague *et al*. 2002) are possible assessment methods of hyperscanning. However, electroencephalography (EEG), with its superior temporal resolution, has been the primary method for studying inter-brain synchronization (Czeszumski *et al*. 2020). The coupling of brain activity between two interacting individuals appears to occur particularly in the temporoparietal and frontal regions, which are known as neural hubs of social cognition (Gvirts and Perlmutter 2020). The setting of the social interaction (e.g. direct exchange), the type of activity (e.g. interactive, cooperative), the interaction partners (i.e. close persons or strangers), and the significance of the interaction for the participants also appear to have a significant influence on inter-brain synchronization (Gvirts and Perlmutter 2020). Finally, higher inter-brain synchronization has been associated with high mutual understanding (Kuhlen *et al*. 2012) as well as high behavioral reciprocity (Nguyen *et al*. 2020a).

Inter-brain synchronization has been observed across multiple frequency bands and topographies, including theta (4–8 Hz; Lindenberger *et al*. 2009, Sänger *et al*. 2012), alpha (8–13 Hz; Dumas *et al*. 2010, Ahn *et al*. 2018), and beta (13–30 Hz; Yun *et al*. 2012, Djalovski *et al*. 2021). By analogy with single-brain oscillatory research, each frequency band is interpreted as reflecting distinct functional processes. In social contexts, theta oscillations are associated with the representation of others’ mental states and action monitoring (Seymour *et al*. 2018, Moreau *et al*. 2020), alpha oscillations with the internal representation of a partner’s cognitive and affective states (Chen *et al*. 2024, Flösch *et al*. 2024), while beta oscillations are associated with sensorimotor coordination and cooperative behavior (Yun *et al*. 2012, Novembre and Iannetti 2021). However, it remains unclear what drives the emergence of inter-brain synchronization: whether it reflects shared sensory input, behavioral coordination, or genuine neural coupling arising from mutual cognitive and affective alignment (Tognoli *et al*. 2020, Moreau and Dumas 2021, Novembre and Iannetti 2021, Holroyd 2022).

Beyond these open questions, converging evidence suggests that inter-brain synchronization is modulated by the social and affective context of interactions, particularly during positive and empathic exchanges involving the mentalizing network. For instance, Djalovski and colleagues examined couples, friends, and strangers during an “empathy task” in which participants shared stressful personal experiences (Djalovski *et al*. 2021). While framed as an empathy paradigm by Djalovski *et al*., such tasks inherently recruit the mentalizing network, as sharing personal narratives requires partners to actively infer and cognitively track each other’s internal mental and affective states. EEG hyperscanning revealed increased inter-brain synchronization in the alpha and beta bands over central and bilateral temporal regions during “empathic” interactions. More recent work further indicates that romantic relationships specifically modulate synchronization processes (Chen *et al*. 2024, Zhou *et al*. 2024). Together, these findings suggest that positive and emotionally meaningful interactions enhance inter-brain synchronization, particularly in the alpha and beta frequency bands.

However, a critical gap remains in the current literature. While existing hyperscanning studies suggest that inter-brain synchronization is modulated by the affective context of an interaction (such as its positive or negative valence), fewer studies have investigated how synchronization changes during tasks that require explicit, cognitive engagement—specifically, active mentalizing. To date, no study has directly investigated the independent contributions of situational interaction valence (positive vs. negative topics) and active cognitive processing (explicitly mentalizing about the partner vs. a non-social control task) within the same dyad. Results might help to understand more about the contextual influence and neural correlates of synchronization in couples. Investigating the specific role of active mentalizing and whether it explicitly drives neural synchronization is of particular importance as understanding provides critical insights: it could suggest that partners do not have to rely solely on a positive emotional mood to achieve neural and psychological alignment. Instead, the active, goal-directed cognitive effort to adopt the partner’s perspective might act as a deliberate buffer, enabling effective dyadic coordination and emotional stabilization even when the immediate situational valence is negative or stressful.

Therefore, the current study aims to explore inter-brain synchronization during naturalistic interactions between couples of parents, with a focus on the effects of interaction valence and mentalization. Couples share a special bond—especially with the challenges of parenting—and interaction and relationship quality are often put to the test. As noted above, negative interactions strain the relationship, whereas mentalizing abilities can be beneficial. First, we wanted to replicate the effect of valence on neural synchronization by analyzing two situational conditions (positive vs. negative interaction topic). Second, we investigated the effect of active mentalizing on inter-brain synchronization using two instructive non-verbal conditions (mentalization vs. counting task). We expected higher levels of inter-brain synchronization in the positive condition than in the negative condition, and in the mentalization condition than in the counting condition.

## Materials and methods

### Participants

Forty-two dyads of parent couples (mother’s age: 35.7 ± 4.1 years; father’s age: 39.2 ± 5.4 years) participated in the study. Dyads had a mean of 1.9 ± 0.6 children with a mean age of 3.5 ± 1.3 years. The mean duration of their relationship was 14.0 ± 4.3 years. Dyads lived together in predominantly middle-income households and had mostly an academic background (average family income between 5000 and 6000 €/month; 18.5 ± 4.0 years of education). From the original sample of 44 dyads, two dyads had to be excluded due to excessive EEG artefacts. General inclusion criteria were age between 18 and 60 years old, German or English as the regular language in the couple, and at least one child between the ages of 1 to 6 years old. Exclusion criteria were left-handedness, brain trauma or severe neurological illnesses, current or past mental disorders, and current psychotropic medication.

### Experimental protocol

Before inclusion in the study, participants were screened via telephone, informed about the study protocol, and gave written informed consent. Father and mother participated together in one laboratory session at the *Core Facility for Neuroscience of Self-Regulation* at Heidelberg University. First, sociodemographic data were recorded, and then participants filled out several questionnaires (see the Questionnaires section). Electrodes for EEG recording were placed on participants’ scalps and faces. After an introduction to the paradigm, a behavioral interaction paradigm (see the Behavioral interaction paradigm section) with simultaneous EEG recording was carried out for about half an hour.

### Questionnaires

The German version of the Interpersonal Reactivity Index was used to measure empathy (Paulus 2009). Relationship satisfaction was measured using the German version of the Relationship Assessment Scale (Hassebrauck 1991).

### Behavioral interaction paradigm

Partners sat facing each other. The paradigm began with a *non-interactive condition*, in which a barrier between the participants blocked their view, and they were asked to look at a fixation cross for 2 minutes. This was followed by a *positive interaction* task: participants were asked to discuss what they agreed about their child’s upbringing and what they valued in each other. After 3 minutes of discussion, a non-verbal condition followed for three minutes, in which participants were asked in randomized order to either mentalize (*mentalizing condition*) or to count the blinks of the partner’s left eye (*counting condition*). In the mentalizing condition both participants were asked to reflect on what the partner is currently experiencing, what he/she thinks about the discussion and their views. This was followed by a further three minutes in the *positive interaction condition*, followed by the remaining *mentalizing* or *counting condition* was administered. The pattern was repeated with a *negative interaction* task, in which participants were asked to discuss disagreements in their parenting and what they disliked about their partner. This was again followed by a *mentalizing* or *counting condition*, then the second part of the *negative interaction* task, and again the remaining *mentalizing or counting condition*. The paradigm ended with another 2-minute *non-interactive condition*. Please also see Fig. 1. After completion, participants were asked, how well they were able to adhere to the task under nonverbal conditions on a scale from 1 (no at all) to 5 (all the time). The average adherence was 4.6.

**Figure 1:**
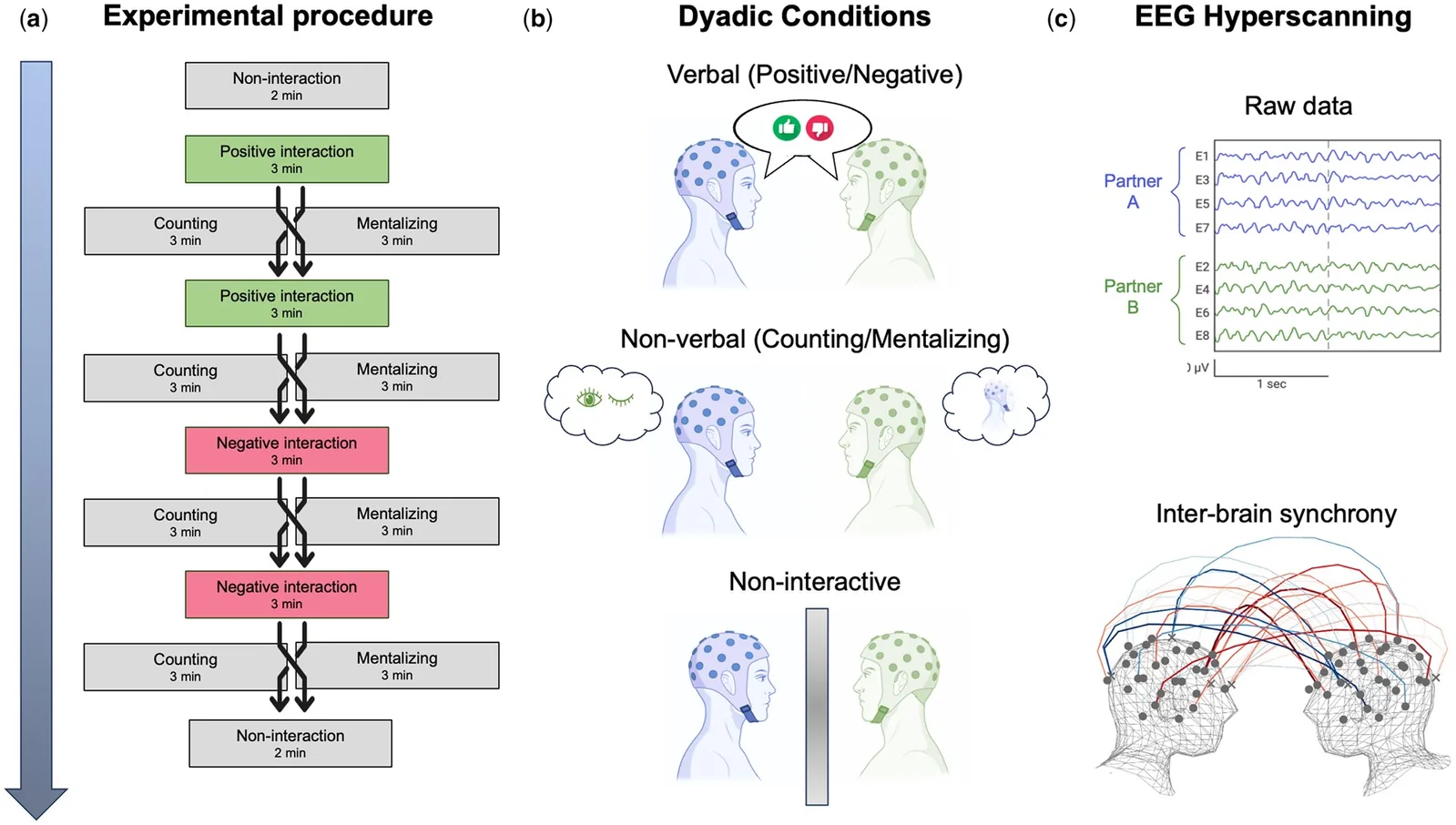
Behavioral interaction paradigm–experimental procedures (a). Dyadic conditions: the paradigm consists of two verbal conditions, in which partners discuss positive or negative parenting topics, two non-verbal conditions, in which parents either try to actively mentalize with the partner or count the partner’s eye blinks, and a non-interactive condition (b). EEG hyperscanning: partners sat opposite each other and EEG raw data was recorded for both participants simultaneously (c).

### Electroencephalogram data acquisition

Neural activity was simultaneously and continuously recorded using EEG while participants engaged in the behavioral paradigm. Data acquisition was performed using BrainAmp DC amplifier with 2x32-channels from the Brain Products Company (Germany). Two Acticap helmets with 32 active electrodes arranged according to the international 10/20 system were used. Additionally, electrooculogram (EOG) was recorded using one electrode on IO for ocular artifact correction. The impedances were kept below 10k Ohm and the reference was placed on TP10 and grounded on Fp1. Data were sampled at 1000 Hz and filtered with a 0.1–500 Hz band-pass filter.

### Behavioral coding

Throughout the entire paradigm, the interaction was videotaped, and videos were macro-coded for individual and dyadic behavior using the couples’ version of the well-validated “Coding Interactive Behavior” manual (Feldman 1998, Djalovski *et al*. 2021). Single behavioral CIB codes were averaged into seven separate composites separately for the positive interaction condition and the negative interaction condition, including sensitivity and intrusiveness separately for mother and father, as well as dyadic reciprocity and dyadic negative states. Sensitivity included the individual codes for “positive affect,” “acknowledging,” “elaborating,” “containing,” “empathy,” “praising,” “warmth and closeness,” and “giving space.” “Negative affect,” “hostility,” and “criticism” were combined for intrusiveness. Dyadic reciprocity included the dyadic coded for “dyadic reciprocity,” “co-regulation synchrony,” and “fluency.” Dyadic negative states were composed of “constriction” and “tension.” Please refer to the manual for detailed information on the codes. Dyadic composites display not only averaged individual codes, but also the dyadic interactional process and dynamic. Coding was conducted by two individual and independent raters who were trained to use the CIB coding system. Interrater reliability on 10 interactions (24%) averaged 87% (intraclass *r* = 0.87).

### Data analyses

Behavioral data were analyzed using IBM SPSS Statistics 29.0 (IBM, Armonk, NY). Dependent *t* tests were used to compare questionnaire data for empathy and relationship quality, as well as behavioral sensitivity and intrusiveness between mother and father. Dependent *t* tests were also used to compare dyadic reciprocity and dyadic negative states between the positive and the negative interaction conditions. Results were considered to be significant at *P* < .05. Cohen’s *d* was used as a measure of effect sizes. Spearman’s correlation was used to explore associations between dyadic reciprocity and dyadic negative states and significant Circular Correlation (CCor) values in the matching conditions. To rigorously account for alpha error inflation across the exploratory analyses, *P*-values for all behavioral correlations were adjusted using the false discovery rate (FDR) method.

To analyze inter-brain synchronization, we compared the conditions of positive versus negative interaction and the non-verbal conditions of mentalizing versus counting separately. EEG data were preprocessed using MNE-Python (Gramfort *et al*. 2013). Data were downsampled to 250 Hz and re-referenced to the average of all electrodes. Before ICA, the data were high-pass filtered at 1 Hz using a zero-phase FIR filter (Hamming window; firwin design). Ocular artifacts were then identified and removed automatically using EOG-based component detection. Post-ICA, the data were notch-filtered at 50 Hz and its harmonics to attenuate line noise and band-pass filtered between 0.1 and 48 Hz using MNE-Python’s default zero-phase FIR filter settings. Continuous data were then epoched into 1-second segments for each experimental condition.

Epoch-level artifact rejection was applied using HyPyP’s AR_local function (Ayrolles *et al*. 2021), which relies on AutoReject (Jas *et al*. 2017), with a peak-to-peak amplitude threshold of 50 µV. Channel-level artifacts were handled within the autoreject procedure, which performs per-epoch interpolation of channels identified as bad within a given epoch (mean = 1.4 interpolated channels per dyad). A union strategy was employed whereby epochs were rejected for both participants in a dyad if marked as artifactual in either participant, ensuring that only time periods with clean data from both participants were retained. On average, 13.5% ± 15.5% of epochs were rejected per condition (range: 7.8%–21.2%), with rejection rates lowest during resting state conditions (7.8%–9.0%) and highest during interaction conditions (15.5%–21.2%). Five dyads were excluded from all analyses due to insufficient clean data across multiple conditions. Dyads with fewer than 30 clean epochs in a given condition were excluded from that condition’s analysis (mean_excluded_dyads_ = 3.33) Clean epochs were then concatenated per condition for further analysis.

Inter-brain connectivity analysis was done using HyPyP, the Hyperscanning Python Pipeline (Ayrolles *et al*. 2021). We used three frequency bands of interest: theta [4–8 Hz], alpha [8–13 Hz], and beta [14–31 Hz]. For each frequency band, we estimated the analytic signal using a multitaper method and calculated the circular correlation coefficient between all inter-brain sensor pairs of a dyad (Moreau *et al*. 2024). CCorr measures the covariance of phase variance between two data streams and is more robust to coincidental synchronization (Burgess 2013) than the phase-locking value. We then used a cluster-level statistical permutation test (Maris and Oostenveld 2007) to contrast CCorr values between dyads and conditions, as implemented in HyPyP. For each frequency band, condition differences were computed at the dyad level and submitted to a dependent-sample permutation *t* test, using a cluster-forming threshold of |*t*| > 2.5. Adjacency across inter-brain sensor pairs was defined using HyPyP’s “*metaconn_matrix_2brains function*,” which extends standard within-brain sensor adjacency to the bivariate inter-brain sensor-pair space by linking pairs sharing at least one spatially adjacent electrode across the two heads. The null distribution was constructed using 5000 permutations of the within-dyad differences. Cluster-level *P*-values reflect the proportion of permutations yielding a cluster mass exceeding the observed value. Frequency bands were tested independently, as each band indexes distinct neural processes, no correction across bands was applied. Consistent with Sassenhagen and Draschkow, cluster-level *P*-values should be interpreted as evidence against the global null hypothesis only, and do not allow inference about the precise location or spatial extent of effects (Sassenhagen and Draschkow 2019).

### Ethical standards

The study was approved by the Ethics Committee of the Medical Faculty at Heidelberg University, Germany, and carried out in accordance with the Declaration of Helsinki of 1975, as revised in 2008. Before inclusion, participants gave written informed consent. They received equal monetary compensation for their participation.

## Results

### Questionnaire data

Mothers showed significantly higher values in self-reported empathy compared to fathers (*M*_mother_ = 55.8 ± 5.5; *M*_father_ = 48.5 ± 6.3; *t*_41_ = 6.64, *P* < .001). The experienced relationship satisfaction between parents did not differ (*M*_mother_ = 4.3 ± 0.6; *M*_father_= 4.3 ± 0.6; *t*_41_ = 0.37, *P* = .715).

### Behavior

Mothers and fathers showed no significant difference in amount of sensitivity (*M*_mother_ = 3.3 ± 0.6; *M*_father_ = 3.2 ± 0.6; *t*_41_ = 1.25, *P* = .219; *d* = 0.42) and intrusiveness (*M*_mother_= 1.6 ± 0.6; *M*_father_ = 1.6 ± 0.5; *t*_41_ = .52, *P* = .584; *d* = 0.48) in individual behavior. Dyads showed significantly more dyadic reciprocity during the positive interaction condition compared to the negative interaction condition (*M*_positive interaction_ = 3.68 ± 1.02; *M*_negative interaction_ = 3.30 ± 0.86; *t*_41_ = 3.45, *P* = .001; *d* = 0.72; Fig. 2a). Dyadic negative states were significantly higher during the negative interaction condition compared to the positive interaction condition (*M*_positive interaction_ = 1.70 ± 0.84; *M*_negative interaction_ = 1.94 ± 0.80; *t*_41_ = −2.18, *P* = .035; *d* = 0.71; F2A).

**Figure 2:**
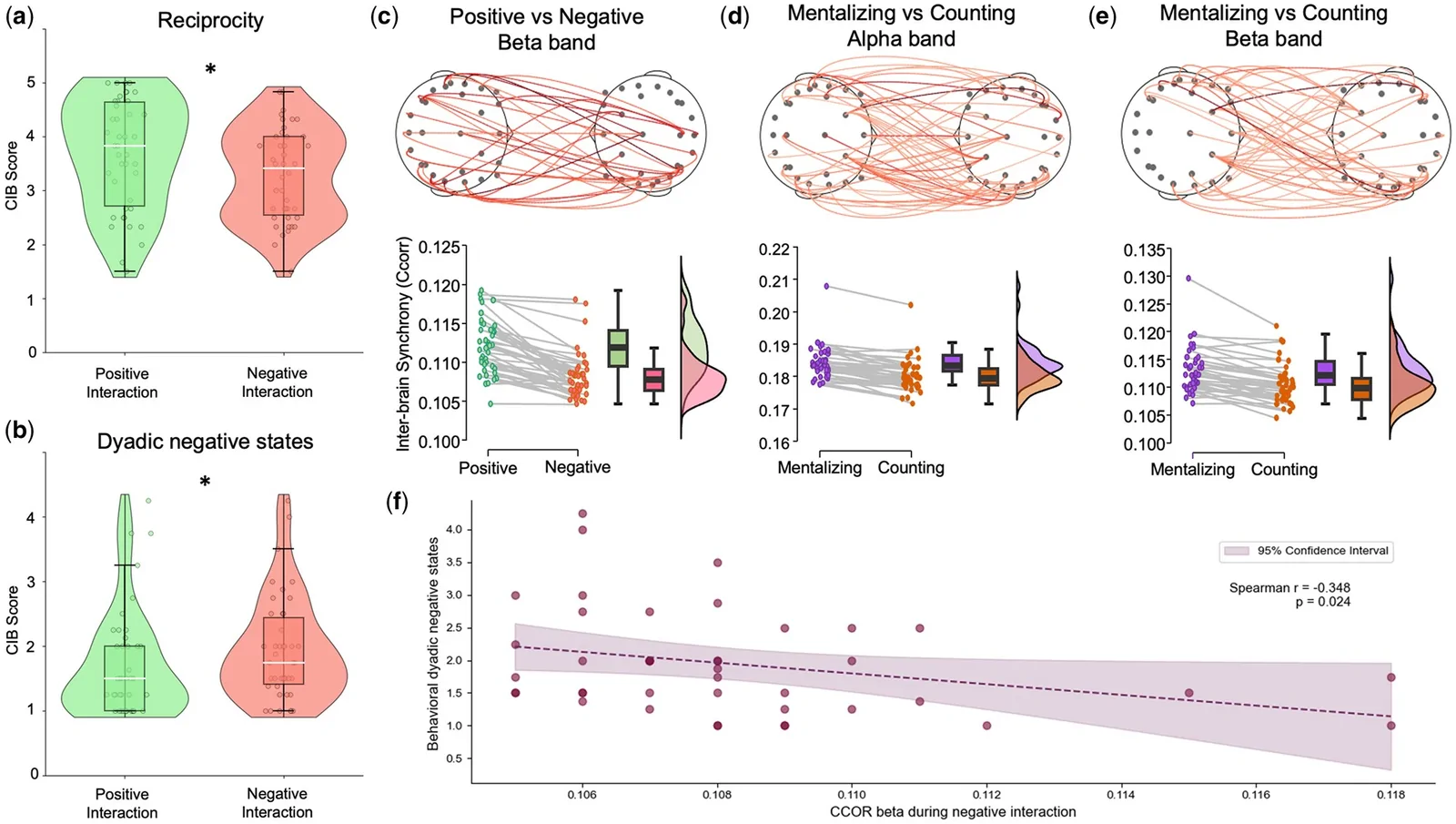
(a, b) Behavioral results. Significantly more dyadic reciprocity during positive compared to negative interaction, and significantly more dyadic negative states during negative compared to positive interaction. (c–e) Results of the cluster-based analysis comparing inter-brain synchronization across conditions. (c) Positive vs. Negative interactions: a significant positive cluster in the beta band (*P* = .0294) indicates greater inter-brain synchronization during Positive compared to Negative interactions. (d) Mentalizing vs. Counting: a significant positive cluster in the alpha band (*P* = .0026) shows increased inter-brain synchronization during Mentalizing compared to Counting. (e) Mentalizing vs. Counting: an additional significant positive cluster in the beta band (*P* = .0276) also reflects stronger inter-brain synchronization in the Mentalizing condition. (f) Significant correlation between behavioral dyadic negative states and CCorr values in the beta band during the negative interaction condition. * = P < .05.

### Inter-brain synchronization results

#### Comparison of positive vs. negative conditions

The cluster-based analysis over the three frequency bands comparing Positive and Negative interactions revealed a significant positive cluster in the Beta band, highlighting increased inter-brain synchronization for Positive compared to Negative conditions (*P* = .0294, *d* = 1.15, see Fig. 2c).

#### Comparison of mentalizing vs. counting conditions

The cluster-based analysis over the three frequency bands comparing Mentalizing and Counting conditions revealed a significant positive cluster in the alpha band (*P* = .0026, *d* = 1.10, see Fig. 2d), and a significant positive cluster in the beta band (*P* = .0276, *d* = 0.69, see Fig. 2e) highlighting increased inter-brain synchronization for Mentalizing compared to Counting conditions in these two frequency bands.

### Associations between dyadic behaviors and inter-brain synchronization

Dyadic negative states correlated significantly with CCorr values in the beta band during the negative interaction condition (*r* = −0.35, *P* = .024; Fig. 2f). No such correlation was found during the positive interaction condition (*r* = −0.13, *P* = .428). For dyadic reciprocity, no significant correlations were found with CCorr values during the positive (*r* = 0.11, *P* = .479) or the negative interaction condition (*r* = 0.17, *P* = .283). None of the tests survived the FDR threshold determined by the Benjamini–Hochberg procedure (critical threshold: *P*_crit_ = .013).

## Discussion

Hyperscanning gives us the opportunity to use a second-person neuroscience approach and to study the neurobiological basis of interpersonal social processes (Redcay and Schilbach 2019). Inter-brain synchronization is understood to support social cognition, improve social alignment, and facilitate predictability in interpersonal interaction (Czeszumski *et al*. 2020, Gvirts and Perlmutter 2020). Additionally, inter-brain synchronization might represent a mechanism of emotional connection between interactional partners and a possible biomarker for interaction quality (Reindl *et al*. 2018, Nguyen *et al*. 2020b). The current study revealed two major findings: (i) inter-brain synchronization was higher during positive compared to negative interactions. Although initial analyses suggested an association between behavioral dyadic negative states and inter-brain synchronization during the negative interaction condition, this correlation did not survive correction for multiple comparisons and should be interpreted with caution as a non-robust trend. (ii) Inter-brain synchronization was higher during active mentalizing than during counting. Additionally, more reciprocity and fewer dyadic negative states emerged in dyadic behavior during the positive versus the negative interaction condition, which confirms that the paradigm elicited the expected results.

Results showing higher inter-brain synchrony during positive compared with negative interactions are consistent with previous research (Hirsch *et al*. 2020, Djalovski *et al*. 2021). Especially interactive and cooperative interactions seem to have a significant influence on synchronization (Cui *et al*. 2012, Mayseless *et al*. 2019, Gvirts and Perlmutter 2020, Carollo *et al*. 2025). Our behavioral analyses confirm that during the positive interaction condition, dyadic reciprocity was higher compared to the negative interaction condition and that dyadic negative states appeared significantly more often during the negative interaction condition compared to the positive interaction condition. Specifically, high mutual understanding (Kuhlen *et al*. 2012) and high behavioral reciprocity (Nguyen *et al*. 2020a) have been associated with elevated inter-brain synchronization in other studies already. Social gaze, positive affect, and a topic of agreement—all factors which support social connectedness and a positive interaction—have also been identified to increase neuronal synchronization (Kinreich *et al*. 2017, Leong *et al*. 2017, Hirsch *et al*. 2020). In the parental relationship, connectedness is especially important to manage the challenges of everyday family life (Pratt *et al*. 2015). In our study, we observed a trend suggesting that increased behavioral dyadic negative states are associated with reduced inter-brain synchronization. This may indicate a decrease in connectedness during disagreement and conflict—a state in which partners tend to focus on their own perspectives rather than actively engaging with the mental states of the other. In fact, it is important to acknowledge that the positive and negative conditions differ along dimensions beyond valence alone. The positive condition likely elicited a stronger affiliative orientation consistent with what Tuomela has theorized as “we-mode,” a collective intentional stance in which individuals act and reason as members of a group rather than as independent agents pursuing separate goals (“I-mode”) (Tuomela 2007, 2013). Under this theory, we-mode engagement promotes shared mental representations and coordinated action, whereas the negative condition, centered on disagreements and dislikes, may have favored a more self-centered, grievance-focused stance with higher emotional arousal. Indeed, Hinvest *et al*. have shown that inter-brain synchrony tracks shared social identity within naturalistic dyads, indexed through affiliative language (Hinvest *et al*. 2025). The observed beta-band IBS difference could therefore be interpreted as reflecting the broader quality of the social exchange rather than valence in a narrow sense. Crucially, the negative condition as implemented did not require genuine mentalizing, as partners could dismiss each other’s positions from a self-centered stance rather than model them, meaning high emotional demand did not translate into the perspective-taking engagement that drives inter-brain coupling. This reflects a naturalistic feature of negative interactions: disagreement and conflict often reduce, rather than recruit, active mentalizing. A complementary mechanistic account is offered by irruption theory as applied to hyperscanning by Froese *et al*. (2024). On this view, heightened subjective involvement in social interaction increases neural entropy, which tends to desynchronize rather than synchronize interacting brains. Reduced beta IBS during the negative condition may therefore reflect not merely disengagement, but the heightened and individually variable neural dynamics that emotionally charged interactions produce. Importantly, irruption theory inverts the naive assumption that greater engagement should always increase synchrony, which is directly relevant to interpreting our valence finding (Froese *et al*. 2024).

Temporoparietal and prefrontal brain regions seem to be especially important for the interactional context (Dumas *et al*. 2020, Czeszumski *et al*. 2022). Restrictions of analysis procedures do not allow us to infer source localization from the topography of the clusters (Sassenhagen and Draschkow 2019). However, at a descriptive level, the coupling of brain activity between interacting partners appears to occur particularly in these neural hubs of social cognition, which could indicate the interrelatedness of biological and behavioral processes (Gvirts and Perlmutter 2020). Descriptive topography indicates that there is increased synchronization in temporo-parietal (alpha, beta) and occipital (beta) clusters in particular during mentalizing. In a rare study on a similar construct to mentalizing, an “empathy giving” task elicited inter-brain synchronization in central and bilateral temporal regions (Djalovski *et al*. 2021). The superior temporal sulcus, posterior cingulate cortex, dorsomedial prefrontal cortex, temporal pole, and TPJ are involved in the mentalizing network supporting attachment in the human brain (Feldman 2017). The mentalizing network appears to facilitate inter-brain synchronization implicating a link between social cognition and alignment processes (Nguyen *et al*. 2020b).

Human relationships rely on mentalizing processes. These underpin attachment formation by understanding the partner’s current cognitive and emotional state, his/her goals, motives, values and concerns in order to be able to align one’s own behavior (Feldman 2017). It is important to study mentalizing as a potential underlying mechanism for inter-brain synchronization, which is suggest to improve social alignment and facilitate predictability—aims which are inherent to mentalizing as well (Hoehl *et al*. 2021). Our study revealed higher inter-brain synchronization during a condition of active mentalizing compared to a counting task. A mentalizing task might require more attention on social cues in the other person compared to the counting task with a stronger alignment in dyadic neural activity. Social attentional processes have been described to foster the alignment of mental states (Lotter *et al*. 2023). The internal representation of another person’s states and actions could also promote inter-brain synchronization. Both tasks were non-verbal, and the interpersonal stimuli were the same (both partners looking at each other) to create comparable conditions. From the results, we cannot tell on which basis the alignment processes took place. Although partners were asked to move as little as possible, subtle synchronization in nonverbal behavior or physiology cannot be excluded (Koul *et al*. 2023).

### Limitations

After much thought, we decided to not counterbalance behavioral interaction conditions in order to prevent carry-over effects of the negative interaction condition. Our experience demonstrated that effects of a negative interaction are often stronger and can have a longer effect on mood and interactive behavior than a positive interaction. Results are also based on a very homogeneous study group and should not be automatically generalized to other socio-economical or age groups. A further limitation concerns the positive versus negative interaction contrast, which likely differs along multiple dimensions simultaneously, including valence, shared identity activation, emotional intensity, and linguistic context. Future studies should aim to dissociate these factors more systematically, for instance by incorporating conditions that orthogonally manipulate valence, sociocognitive engagement, and the affiliative content of language. Finally, the study was not pre-registered, and the analyses reported here should therefore be regarded as exploratory rather than confirmatory.

## Conclusion

This study demonstrates that inter-brain synchronization is enhanced during positive interactions and active mentalizing, establishing it as both a phenomenon of social cognition and a potential biomarker for relationship quality. Mentalizing and prosocial behavior seem to drive inter-brain synchronization in the interaction of couples. These results support the existing research, which characterizes inter-brain synchronization as a neural correlate of social cognition and a biomarker for interaction quality (Gvirts and Perlmutter 2020, Nguyen *et al*. 2020a, Moreau and Dumas 2021). They also confirm hyperscanning represents a paradigm shift in social neuroscience, revealing that human interaction fundamentally operates through dynamically coupled neural systems rather than isolated individual processes (Dumas 2011, Kelso *et al*. 2013).

By integrating behavioral and neurobiological methods, our approach reveals that inter-brain synchronization serves as a quantifiable biomarker of social-cognitive engagement and relationship quality. This innovative study in which partners talk about their children shows the importance of mentalizing and positive exchange. Raising a child together is a challenge that requires a close connectedness and coordination in order to be successful. These findings have direct clinical implications: interpersonal synchrony measures could enable early detection of social dysfunction and guide targeted interventions across developmental and clinical populations, but also measure therapeutic alliance (Dumas 2022, Adel *et al*. 2025). Future research must establish mechanistic links between behavioral dynamics and neural synchronization by integrating multi-scale analyses, combining micro-behavioral analyses and physiological data in various and larger contexts, to elucidate the fundamental principles governing interpersonal neural coupling.

## Data Availability

Due to the nature of this research, participants of this study did not agree for their data to be shared publicly, so supporting data are not available.
